# Integrative profiling of Epstein–Barr virus transcriptome using a multiplatform approach

**DOI:** 10.1186/s12985-021-01734-6

**Published:** 2022-01-06

**Authors:** Ádám Fülöp, Gábor Torma, Norbert Moldován, Kálmán Szenthe, Ferenc Bánáti, Islam A. A. Almsarrhad, Zsolt Csabai, Dóra Tombácz, János Minárovits, Zsolt Boldogkői

**Affiliations:** 1 Department of Medical Biology, Albert Szent-Györgyi Medical School, University of Szeged, Somogyi B. u. 4., Szeged, 6720 Hungary; 2 Carlsbad Research Organization Ltd., Szabadság u. 2., Újrónafő, 9244 Hungary; 3 RT-Europe Research Center, Vár tér 2., Mosonmagyaróvár, 9200 Hungary; 4 Department of Oral Biology and Experimental Dental Research, University of Szeged, Tisza Lajos krt. 64, Szeged, 6720 Hungary

**Keywords:** Epstein–Barr virus, Herpesvirus, Transcriptome, Transcript isoform, Splice variant, Transcription start site, Transcription end site, Long-read sequencing, Nanopore sequencing, PacBio sequencing

## Abstract

**Background:**

Epstein–Barr virus (EBV) is an important human pathogenic gammaherpesvirus with carcinogenic potential. The EBV transcriptome has previously been analyzed using both Illumina-based short read-sequencing and Pacific Biosciences RS II-based long-read sequencing technologies. Since the various sequencing methods have distinct strengths and limitations, the use of multiplatform approaches have proven to be valuable. The aim of this study is to provide a more complete picture on the transcriptomic architecture of EBV.

**Methods:**

In this work, we apply the Oxford Nanopore Technologies MinION (long-read sequencing) platform for the generation of novel transcriptomic data, and integrate these with other’s data generated by another LRS approach, Pacific BioSciences RSII sequencing and Illumina CAGE-Seq and Poly(A)-Seq approaches. Both amplified and non-amplified cDNA sequencings were applied for the generation of sequencing reads, including both oligo-d(T) and random oligonucleotide-primed reverse transcription. EBV transcripts are identified and annotated using the LoRTIA software suite developed in our laboratory.

**Results:**

This study detected novel genes embedded into longer host genes containing 5′-truncated in-frame open reading frames, which potentially encode N-terminally truncated proteins. We also detected a number of novel non-coding RNAs and transcript length isoforms encoded by the same genes but differing in their start and/or end sites. This study also reports the discovery of novel splice isoforms, many of which may represent altered coding potential, and of novel replication-origin-associated transcripts. Additionally, novel mono- and multigenic transcripts were identified. An intricate meshwork of transcriptional overlaps was revealed.

**Conclusions:**

An integrative approach applying multi-technique sequencing technologies is suitable for reliable identification of complex transcriptomes because each techniques has different advantages and limitations, and the they can be used for the validation of the results obtained by a particular approach.

**Supplementary Information:**

The online version contains supplementary material available at 10.1186/s12985-021-01734-6.

## Introduction

Epstein–Barr virus (EBV, human gammaherpesvirus 4), is a member of the *Gammaherpesvirinae* subfamily within the family *Herpesviridae* [1]. Spreading predominantly via saliva, EBV is highly prevalent in human populations [2]. EBV plays a role in the pathogenesis of Burkitt’s lymphoma and other lymphomas, and it is also involved in the development of nasopharyngeal carcinoma and a subset of gastric carcinomas [3, 4]. EBV is classified as a Group 1 carcinogenic agent in humans [5]. Additionally, EBV reactivation has been suggested to be one of the main causes of long COVID symptoms [6]. Primary EBV infection in early childhood is typically mild or symptomless. Later in life, however, it may cause infectious mononucleosis (IM, glandular fever), a lymphoproliferative disease accompanied with pharyngitis, tonsillitis, fever and lymphadenopathy. In the majority of cases, IM is a self-limiting disease, due to a vigorous T cell response directed to EBV-infected, proliferating B cells expressing viral antigens [7]. Although B cells are the primary targets of EBV, the virus also replicates in oropharyngeal epithelial cells. Both B cells and epithelial cells are capable to produce new EBV particles that carry linear, double stranded viral genomes.

After the primary infection, the virus establishes lifelong latency in memory B cells [8]. In latently infected cells, only a limited set of viral genes are expressed from the circular, episomal, chromatinized EBV genomes. In addition to memory B cells, EBV-associated lymphoma and carcinoma cells and in vitro immortalized lymphoblastoid cell lines (LCLs) can also carry latent EBV genomes. The host cell epigenetic machinery interacts with the viral episomes and the activity of latent EBV promoters is regulated by the epigenetic marks deposited by host enzymes to the transcriptional control sequences of the viral genome [9]. In latently infected cells, the viral episomes attach to the nuclear matrix at *oriP*, the latent origin of EBV replication and replicate once per cell cycle with the help of the host DNA synthesis machinery [10]. A variety of signals are capable to disrupt latency and induce EBV lytic reactivation both in vitro and in vivo [11, 12]. Induction of EBV lytic replication results in a change from the restricted, latent pattern of EBV gene expression to successive transcription of immediate early (IE), early (E) and late (L) EBV genes.

The IE gene products designated BZLF1 and BRLF1 are transactivator proteins switching on the transcription of early genes [13, 14]. The E gene products include, among others, a core set of lytic replication proteins shared by *Herpesviridae* [15]. Lytic EBV DNA synthesis occurs in the replication compartments within the host cell nuclei [16]. In contrast to the replication of latent episomes, this unlicensed, exponential amplification of the viral genome is initiated at one of the two copies of *oriLyt*, the lytic replication origin of EBV DNA synthesis [10]. It has been suggested that during productive replication the IE and E EBV genes are transcribed from chromatinized templates, whereas unchromatinized, unmethylated templates are used for the transcription of L genes encoding structural proteins of the virion [17]. EBV late RNA transcription is aided by the viral preinitiation complex [18]. After the synthesis of viral structural proteins, epigenetically naïve, unmethylated, linear dsDNA molecules are packaged into EBV virions [17, 19]. These linear genomes undergo circularization, chromatization and epigenetic modification in the newly infected host cells.

Initial studies indicated that all of the viral genes charted on the approximately 170 kb EBV genome are actively transcribed during the lytic cycle [20–23]. Recent studies of the viral transcriptome revealed, however, a more complex pattern of viral gene expression after the disruption of EBV latency in various cell lines. It turned out that lytic cycle transcription is bidirectional, and that many newly identified transcribed regions do not code for proteins [24–27]. These data suggest that hundreds of viral long noncoding RNAs (lncRNAs) may be generated during productive EBV replication. In addition, novel splicing events further increase the diversity of EBV transcriptome expressed during the productive replication [28].

Next- and third-generation sequencing technologies have proved to be highly efficient in characterizing the structural and kinetic aspects of transcriptomes [29]. The Illumina platform is able to produce high data coverage and base accuracy, which allow the identification of splice sites, transcriptional end sites (TESs), and RNA editing [30, 31]. However, the short read lengths lack the information needed to detect the alternative transcriptional start sites (TSSs), splice isoforms, embedded transcripts, and parallel transcript overlaps. Long-read sequencing (LRS) technologies developed by Pacific Biosciences (PacBio) and Oxford Nanopore Technologies (ONT) allow the detection of full-length RNA molecules at the price of a lower throughput and higher sequencing error rates [32, 33]. LRS is more efficient than short-read sequencing (SRS) for determining 5′- and 3′-UTR isoforms, splice variants, the long RNA molecules, including the polygenic transcripts, as well as the overlapping and embedded transcripts [34–36]. Compare to ONT platform, the major limitation of PacBio and Illumina approaches is that they are inefficient in reading nucleic acid sequences within the range of 200–800 nucleotides. Furthermore, the ONT platform can be used for native RNA sequencing [37]. Although the ONT platform works with a relative high error rate, this does not a present a problem in transcriptome research for well-annotated genomes even in the case of low coverage. Applying an integrated approach, including LRS and SRS platforms in conjunction with other supplementary techniques can eschew the deficiencies of each technique.

Earlier studies based on Illumina short-read sequencing (SRS) [18, 24, 26, 38] and Pacific Biosciences (PacBio) RS II sequencing [26] identified a large set of EBV transcripts. However, SRS is not optimal for disclosing the transcriptome complexity [39, 40] and RS II sequencing has also a limitation for the detection of transcripts falling into a certain size interval [36, 41].

In this work, we analyzed the EBV lytic transcriptome using the Oxford Nanopore Technologies (ONT) MinION sequencing platform, which is suitable to provide a complete picture on the viral transcriptomic architecture [42–45], excluding small non-coding RNA molecules, such as micro RNAs. Both LRS approaches are able to determine the splice sites, the transcriptional start sites (TSSs) and the transcriptional end sites (TESs) without the involvement of additional techniques. In this report, we apply the LoRTIA [46] software suit developed in our laboratory for the identification of RNA molecules and filtering out the artefactual transcripts generated by false priming, template switching, sequencing, etc.

## Results

### Multiplatform profiling of the EBV transcriptome

In this work, we analyzed the lytic EBV transcriptome using our novel amplified and non-amplified ONT sequencing dataset, as well as transcriptomic data generated by others using PacBio RSII [26] and Illumina [25–28, 40, 41] platforms. PCR is used for the amplified techniques, whereas no PCR is applied for the non-amplified approaches. ONT and PacBio data were used for the identification of full-length RNA molecules, whereas Illumina CAGE-Seq and Poly(A)-Seq data were used for the validation of TSSs, TESs and splice site. The data obtained by this multiplatform approach were integrated for the detection of novel EBV transcripts and verification of already described RNA molecules. LoRTIA program (developed in our laboratory; https://github.com/zsolt-balazs/LoRTIA) was used for the annotation of novel transcripts and filtering out the spurious transcripts. We used stringent criteria for the identification of viral RNAs (see below). Oligo(dT)-primed amplified and non-amplified (direct) cDNA libraries were generated from eight consecutive lytic time points. However, due to the low coverage, especially at the early time-points, kinetic analysis was not feasible from this dataset. A total of 22,358 non-amplified and 54,271 amplified reads mapped to the viral genome with an average mapped read length of 838.66 nts (σ = 701.5) and 1098.43 nts (σ = 758.28) respectively. The numbers of reads obtained by other techniques were as follows, PacBio: 104,469, Illumina Cage-Seq: 3,344,162 and Illumina polyA-Seq: 93,817,061. The detailed statistics of the sequencing can be found in Additional file 1. We also generated random hexamer-primed amplified library from pooled samples and sequenced them on the MinION platform**.**

### Transcripts ends and alternative ends

**Fig. 1:**
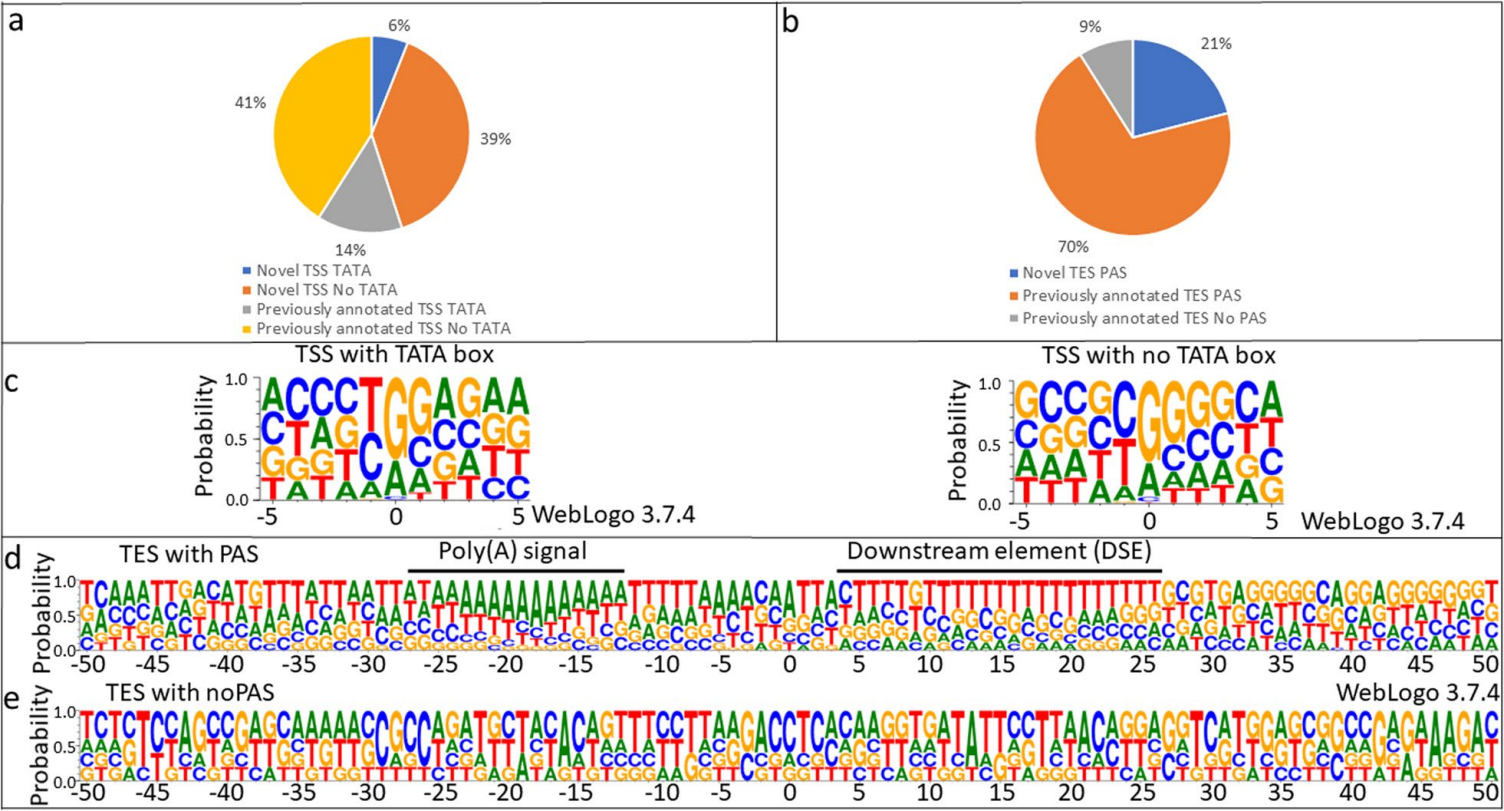
Sequence motifs and proportions of TSSs and TESs. Distribution of TATAA and non-TATAA promoters (**a**). Distribution of TESs without a poly(A) signal (PAS) and with a PAS signal (**b**). Sequences surrounding the (c) TSS contain a G-rich initiator region for TSSs with a TATA box. TSSs lacking a TATA box have G-rich + 1 and + 2 positions. TESs with a PAS have a canonical A-rich cleavage site and a canonical GU-rich downstream element (DSE) (**d**), while those without a PAS have a C-rich cleavage with no recognizable DSE (**e**)

This study detected a total of 398 putative TSSs (Additional file 2). We used CAGE-Seq, ONT-MinION and PacBio datasets [26] for the validation of our TSSs. A TSS was accepted if it was present in at least two of our techniques or in one of our techniques and either in the CAGE-Seq or in the PacBio dataset. This stringent filtering resulted in a total of 322 TSS of which 145 are novel (Fig. 1a). We identified all of the TSSs which were detected by CAGE-Seq, and 4.66% (14 out of 322) of the TSSs were undetected by CAGE-Seq. Upstream TATA boxes were identified for 20% of the TSSs at an average distance of − 31.43 nts (σ = 3.31). The nucleotide composition analysis of these start sites revealed a G-rich initiator region (Fig. 1c). Sixty-two GC boxes were identified with a 64.70 nt average distance from the TSSs. The average distance of the identified 17 CAAT boxes from the TSSs is 110.23 nts. Both the GC and CAAT boxes are promoter consensus elements, which bind specific transcription factors (SP1 and NF-1, respectively). The GC box consensus sequence is as follows: GGGCGG. This sequence and is located within 100 nucleotides upstream of the TSSs. The CAAT box consensus sequence is CAATCT, which is located ~ 75 nucleotides upstream of the TSSs.

A total of 65 putative transcription end sites (TESs) were detected using the LoRTIA software suite (Additional file 3). A TES was accepted if it was present in at least two of our techniques or in either one of our techniques and in the PacBio dataset⁠, or the PA-seq dataset [26, 28, 41, 43]. This analysis resulted in the detection of 57 TESs of which 12 are novel (Fig. 1b). Polyadenylation signals (PASs) were identified for 89% of the TESs at an average distance of − 24.51 nts (σ = 6.99). TESs with a PAS showed an A-rich cleavage site and a G/T-rich downstream region. These sequences are similar to those of the mammalian cleavage and polyadenylation motifs [50] (Fig. 1d). TESs lacking a PAS showed a ACCTC sequence near the cleavage site (underlined) and a TTATT sequence between + 11 and + 15 positions (Fig. 1e), the latter being a variant of the termination signal of genes transcribed by RNAPIII [51], a polymerase specialized in transcribing rRNAs and tRNAs. However, it has been described as a terminator for the VA RNA gene of avian adenovirus CELO [39], suggesting the possibility of incidental transcription by RNAPIII for protein coding genes.

**Fig. 2:**
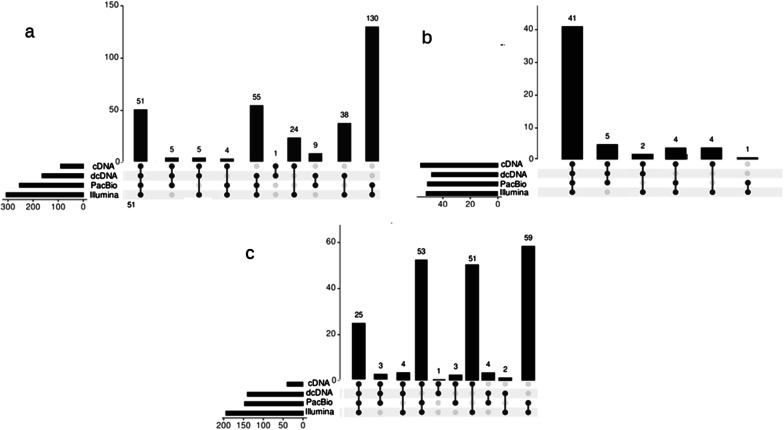
UpSet diagram for the quantitative distributions of TSSs (**a**), TESs (**b**) and introns (**c**) identified by a multiplatform approach, including ONT MinION, PacBio Sequel and Illumina platforms. Horizontal bar graphs: The horizontal bar graph shows the number of **a** TSSs, **b** TESs, and **c** introns detected by the given sequencing approaches. The vertical bar graphs show the number of **a** TSSs, **b** TESs, or **c** introns (y axis) detected in different combinations of sequencing methods (x axis). Black dots indicate the presence of transcripts in the particular experiments

We used our validated TSSs (Fig. 2a), TESs (Fig. 2b) and introns (Fig. 2c) for the annotation of transcript isoforms. This resulted in a total of 351 polyadenylated transcripts (Additional file 4; Table 1). We compared the transcripts annotated in our work with those of reported by O’Grady et al. [26] (Additional file 5) and obtained that 108 transcripts were identified in both studies, while 185 transcripts were detected by only O’Grady and colleagues and 241 transcripts in only our study. The discrepancy between the two studies is explained by two reasons: the average read-length were higher in the PacBio (1176 nts) than in the ONT (963 nts) data; and that we applied very strict criteria for accepting reads as true transcripts.

**Table 1:** The number of previously annotated and novel EBV transcripts

Transcript types	Previously annotated	Novel
Monocistronic transcripts	7	15
5′-UTR isoforms	23	104
Multigenic transcripts	14	47
Isoforms with alternative termination	1	7
Complex transcripts	2	6
Splice isoform	31	42
Non-splice isoforms	2	5
Putative protein-coding transcripts	25	47
Non-coding transcripts …	7	21
… of which antisense transcripts	1	1

Some transcripts are represented by only a single read, which is below the threshold of detection of LoRTIA. Because of their low abundance their TSS is uncertain, hence we denote these as putative transcript isoforms. We detected a total of 52 putative transcripts, including 33 that are longer than any other overlapping transcripts (Additional file 6), but we think that a higher data coverage would reveal a much larger population of these transcripts.

A former LRS study disclosed a large diversity of TSSs and TESs for several EBV genes [26]. Here, we identified 104 novel 5′-UTR isoforms of which 47 have longer and 57 shorter 5′-UTRs. The CAGE-Seq data analysis validated 98% of our longer TSSs and 92.98% of shorter TSSs isoforms.

The 5′-UTRs can regulate translation through their secondary structures [53], upstream AUGs (uAUGs), or upstream ORFs (uORFs) [54, 55].

Watanabe and colleagues [56] analyzed the impact of two uORFs upstream of BGLF3.5 ORF on the translation of BGLF4, a protein kinase involved in replication and nuclear regress [57], and found that point mutations in the two disruption of uORFs (duORFs) had no effect on protein levels of BGLF4.

**Fig. 3:**
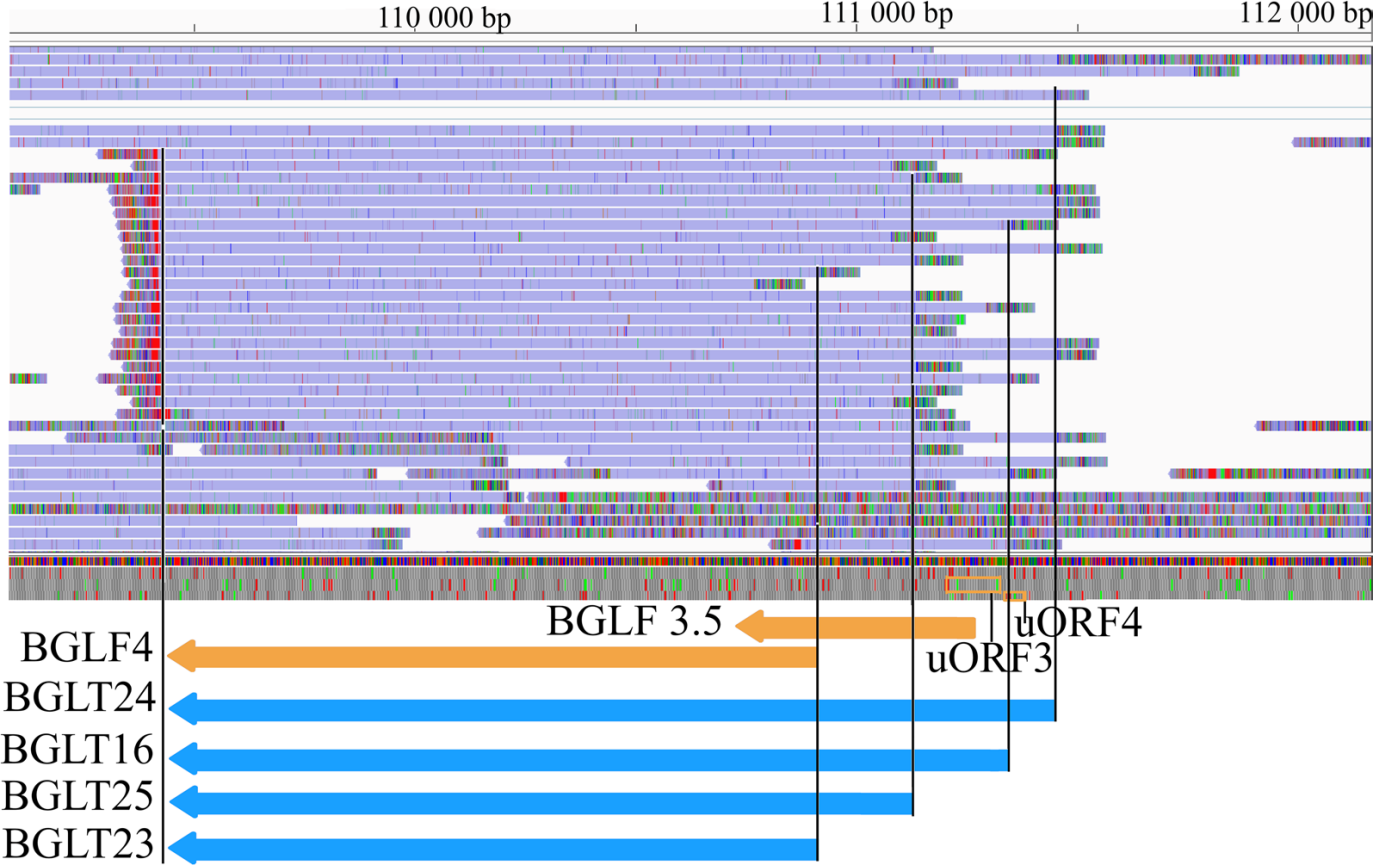
Transcripts overlapping BGLF4 and BGLF3.5 ORFs and their uORFs. Yellow arrows indicate the BGLF4 and BGLF3.5 genes, while the blue arrows show the transcripts encoded by these genes (BGLT24, BGLT16, BGLT25, BGLT23). Two upstream ORFs (uORF3 and uORF4) are also indicated. Purple bars indicate the reads obtained by the ONT MinION sequencing. Rectangular lines show the transcript ends

The most abundant transcript of the BGLF3.5-BGLF4 cluster is BGLT16, a bicistronic mRNA consists of wild-type uAUG upstream (Fig. 3). We detected two short 5′-UTR isoforms of this transcript (BGLT23 and BGLT25) carrying solely the BGLF4 gene. This RNA molecule lacks the duORFs mutated by Watanabe and co-workers [56]. Furthermore, BGLT24, a longer 5′-UTR isoform of BGLT16 contained additional wild-type uAUGs and uORFs upstream from the point mutations created by Watanabe and coworkers (Fig. 3).

**Fig. 4:**
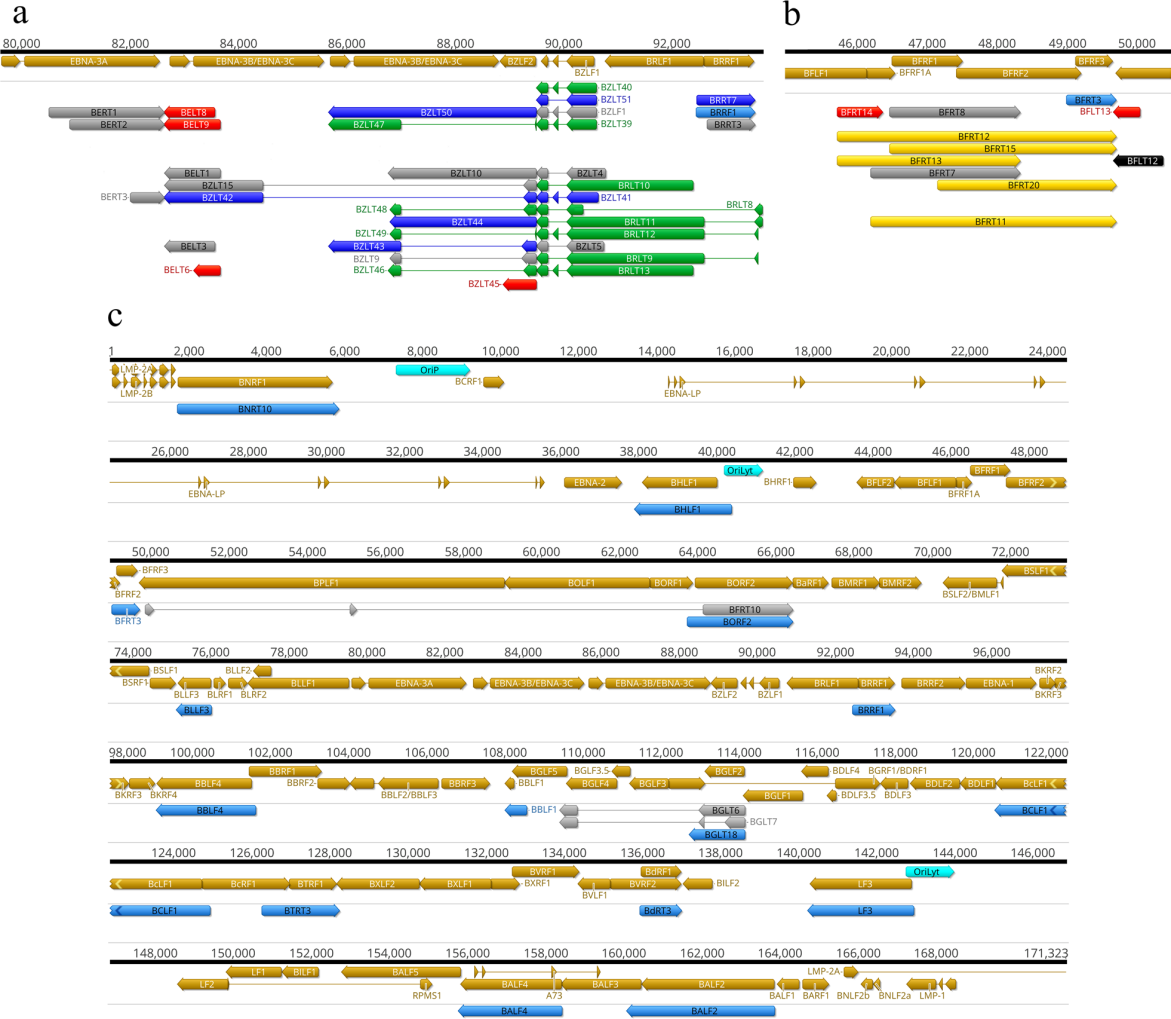
Novel alternative polyadenylation sites and monocistronic mRNA-s with canonical ORFs. **a** Seven annotated transcripts with alternative polyadenylation sites. **b** Genomic region of BFRF3 where previously transcripts were not annotated, we identified 1 novel monocistronic and 4 polycistronic transcript. **c** Novel monocistronic mRNAs where only spliced versions have previously detected. Color codes: brown arrows: ORFs; aqua rectangles: replication origins; grey: formerly annotated transcripts; light blue: novel monocistronic transcripts; yellow: novel polycistronic transcripts; red: non-coding transcripts; black: 5′-truncated transcripts; dark blue: TSS and TES isoforms

Our analysis disclosed 7 isoforms with alternative polyadenylation sites of which 4 are novel. Intriguingly, all of them are located within the same 5 kb region, BZLT42, BZLT43 BZLT44 and BZLT50 are 3′-UTR isoforms of the BZLF2, whereas BELT6, BELT8 and BELT9 are isoforms of the BELT1 transcripts (Fig. 4a). As a consequence, a 10 nt-long convergent overlap is formed between BZLT44, BELT8, BELT9 and BERT3 (Additional file 4).

### Novel monocistronic mRNAs with canonical ORFs

Here we report the detection of 15 novel monocistronic transcripts (Additional file 2). We identified unspliced BNRT10, BHLF1, BORF2 and BGLT18 transcripts of which only spliced versions have previously been detected [26, 28] (Fig. 4c). We also discovered 10 novel monocistronic transcripts with full-length ORFs of which only shorter isoforms with incomplete ORFs (they lack in-frame AUG) have been previously described [26]. Transcripts have not yet been annotated in the genomic region of BFRF3, although studies demonstrated this region transcriptionally active [58, 59]. We identified BFRT3 with a novel fully overlapping the BFRF3 ORF and ending in a novel terminus (Fig. 4b). (Additional file 2).

### Splice junctions and introns

Reverse transcription and PCR may generate gaps in the cDNAs through template-switching (TS) events, which can lead to false intron annotation. The LoRTIA software suite is capable to eliminate these artifacts by detecting the absence of splice junction consensuses or the presence of repeat regions which favors TS. We detected 205 introns using the LoRTIA tool kit and applying the criterions that a putative intron has to be present in at least 2 of our techniques or in one of our techniques and either in the Illumina or in the PacBio dataset. A canonical GT/AG splice junction consensus was present in every identified intron (Additional file 7).

### mRNAs with altered coding potential

**Fig. 5:**
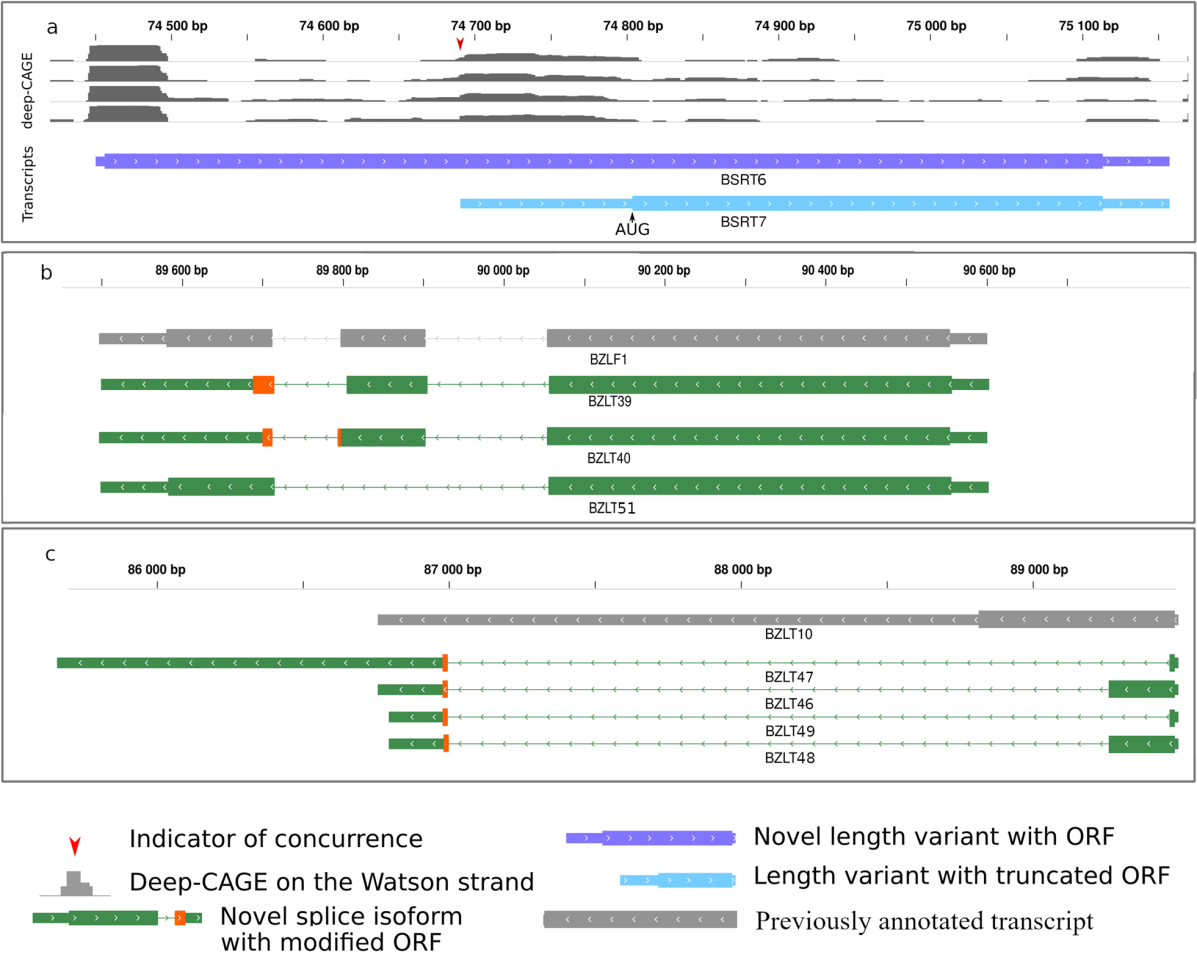
Transcripts with altered coding potential. **a** Transcript isoforms with TSSs located within the canonical ORFs of the host genes in many cases contain in-frame truncated ORFs. The gray histogram illustrates the CAGE-Seq data generated by O ‘Grady and colleagues [26]. **b** Alternative splicing of the BZLF1 transcripts results in nonsense termination of the ORFs highlighted by the orange wide rectangles and in deletion of the exon in BZLT51 transcript that does not cause frameshift but an ORF with deleted nucleotides. **c** Splicing of the unspliced BZLT10 leads to the deletion of a large portion of the ORF and results in an intergenic stop codon (first exon shown by green, while the second exon shown by the orange wide rectangles). This figure shows examples, and not a summary of all data

We detected several transcripts with truncated 5′-ends having the same TESs as the host mRNAs. These short RNA molecules lack the canonical ORF, but contain downstream in-frame AUGs, and thus may code for N-terminally truncated proteins [60]. We report 72 such RNA molecules of which nineteen are novel. Seventy-two TSSs of these transcripts were confirmed by the CAGE-Seq dataset (Fig. 5a). Besides the alternative transcription initiation within a gene, alternative splicing is also able to produce transcripts with altered coding potential if splicing occurs within the ORF. Forty-two novel splice isoforms and 5 unspliced versions of previously annotated spliced transcripts were detected in this analysis. Nineteen transcripts contained introns within the ORFs. Among these, 9 frame-shifting, 2 nonsense terminations (through intron retention leading to premature stop codon) (Fig. 5b), 4 ORFs with deleted amino acids (in-frame deletion) (Fig. 5b) and 4 intergenic terminations (Fig. 5c) were identified. These latter transcripts contain the regular AUG and a new stop codon in an intergenic position.

The coding potential of these transcripts was evaluated using the Coding-Potential Assessment Tool (CPAT) with default settings [61]. CPAT uses four parameters to estimate the coding potential. The first parameter is the maximum ORF length, the second is the coverage of the ORF, and the third is the Fickett score, which separates the mRNAs from ncRNAs, which is based on codon usage and nucleotide composition. The fourth factor is the hexamer score, which gives the difference between the coding and non-coding sequences (+ coding, + non-coding). Sensitivity gives the ratio of false negatives. Specificity estimates the incidence of false positives. Accuracy is a combination of the sensitivity and specificity.

First, we evaluated CPAT’s performance by running the software on known coding transcript isoforms and isoforms lacking an ORF. This resulted in a sensitivity of 0.87, a specificity of 0.93 and an accuracy of 0.89, suggesting the usability of the default parameters on our dataset. Then we calculated the coding probability of 5′-truncated, alternatively spliced and unspliced transcript isoforms. CPAT analysis led to the result that 9 of the 5′-truncated isoforms, and all—except the ORFs of the 4 splice isoforms with intergenic termination—may have coding potential (Additional file 8). Therefore, these latter transcripts are considered as non-coding RNAs (ncRNAs). To investigate the homology of proteins encoded by alternatively spliced transcripts, we queried the translation of the altered ORFs to the NCBI non-redundant protein database using protein BLAST. BNRT11 and BNRT12 have the first 21 amino acids of the BNRF1 ORF but ends in a stop codon directly after the first splice acceptor position. For BHLF2 splicing results in frame shifting. The first 77.38% of the ORF is identical to the BHLF1 ORF, whereas the amino acids following the splice acceptor position show no similarity to other proteins in the database. The splice acceptor position of BSLT12, BSLT18 and BSLT21 differ from the splice acceptor of the main isoform (BSLT13). This results in altered amino acids following the acceptor position, which do not match any other protein in the database. BZLT46 and BZLT48 encode the first 75 amino acids of BZLF2 ORF, whereas the following amino acids and the stop codon is spliced from the transcript. Thus, the altered ORF continues and ends in the second exon of these transcripts, with the amino acids following the acceptor position showing no similarity to proteins in the database. The second splice donor position of BZLT39 and BZLT40 differs from that in the main isoform (BZLF1) resulting in frame shifting, 7 amino acids and a stop codon follows after the corresponding splice acceptor. In BZLT51 the second exon of the BZLF1 are deleted this is a 35 amino acid shortening, but the deletion doesn’t cause any frame shifting. BART17 a splice isoform of the BART transcripts retains the first and the third introns. The first intron encompasses an in-frame stop codon. The resulting altered protein shows partial homology with the first exon of the a73 ORF. The putative proteins of BZLT47 and BZLT49 and BRLT8 show no homology to any other entry in NCBI (Additional file 8).

### Non-coding transcripts

Transcripts lacking an ORF longer than 10 amino acids were categorized as non-coding. In this part of this study, we detected 2 short ncRNAs (sncRNAs; shorter than 200 nucleotides) and 19 lncRNAs (longer than 200 nts). Fourteen of the lncRNAs are 5′-truncated, whereas three of them (BFRT14, BLRT9 and BZLT45) are 3′-truncated isoforms of previously annotated RNAs. BLRT9, a lncRNA starts in the same position as BLRT5 but is terminated 490 nts downstream detected by both our analysis and the Illumina PA-Seq. BLRT9 overlaps the BZTL and BELT region in antisense orientation.

### Ori-associated transcripts

**Fig. 6:**
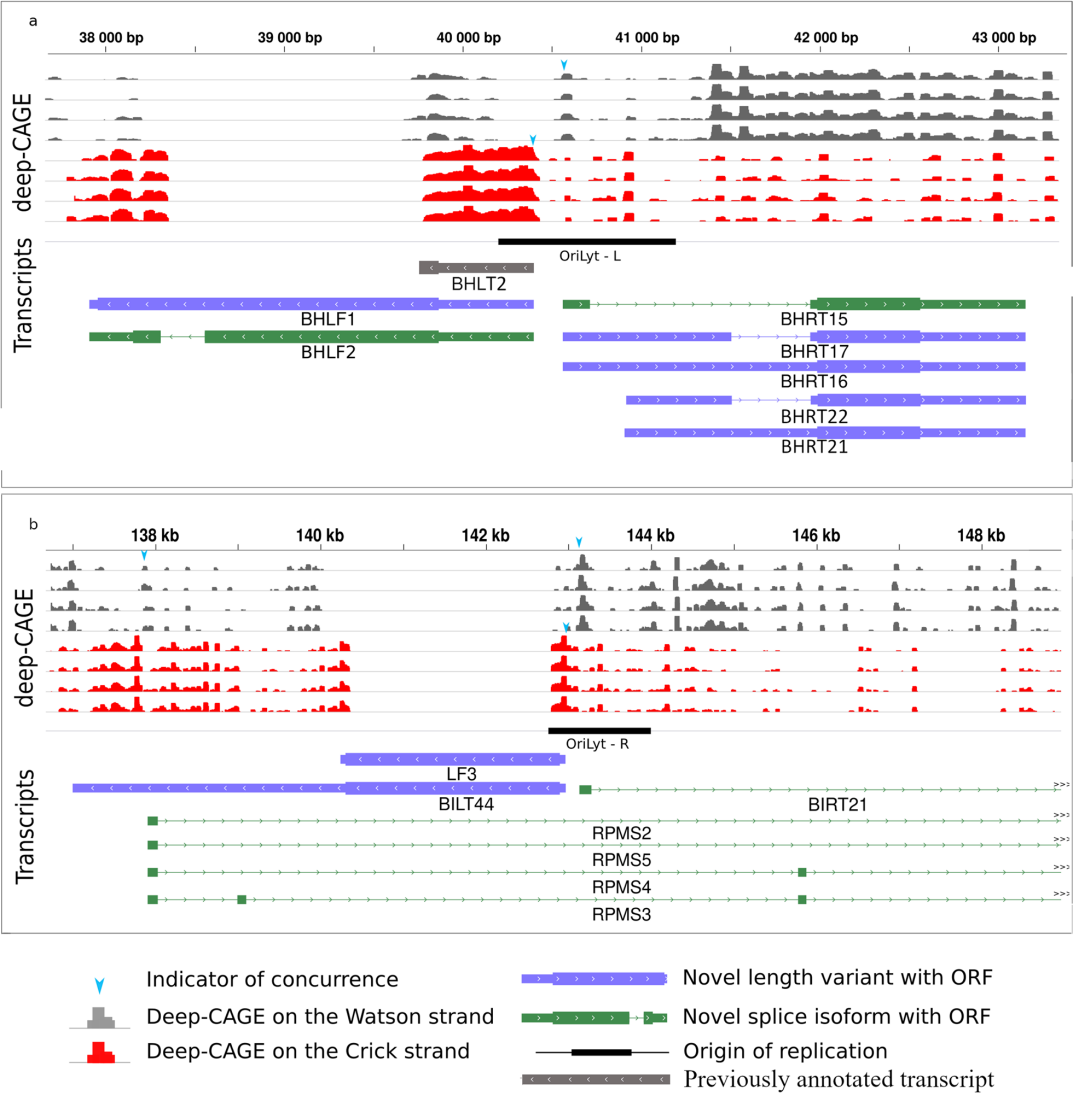
Novel Ori-overlapping RNAs. **a** Three novel length and two novel splice transcript isoforms are initiated in the left lytic Ori region. The CAGE-Seq data from O ‘Grady et al. [26] is represented by the histograms with gray reads mapping to the positive, while red are reads mapping to the negative strand. Light blue arrow heads show matching between the CAGE-Seq data and our TSS data. **b** Two novel length and five novel splice isoforms initiated or overlapping the right lytic Ori region

Eukaryotic replication origins are generally associated with coding and non-coding transcripts [62, 63], Ori-overlapping transcripts were previously demonstrated in alpha- [35, 45], beta- [64, 65] and gammaherpesviruses [66]. The EBV genome possesses two lytic (OriLyt) and a single latent (OriP) origin of replication. The left OriLyt has been shown to overlap the splice isoforms of BWRT and BCRT, whereas BHLT2 starts within this Ori [26]. The genomic region containing OriP also shows transcriptional activity: several TSSs of various ncRNAs located within the Ori [25] and one long 5′-UTR transcript isoform of BCRF1 gene the BCRT3. We detected 9 novel isoforms of Ori-associated RNAs, all of which were initiated within one of the lytic replication origins. BHLF1 and 2 transcripts are encoded by the bhlf1gene. BHRT15, 16, 17, 22, and 22 transcripts are splice and 5′-UTR isoforms and are encoded by the brf1 gene (Fig. 6a). The LF3 transcript starts in the right OriLyt, region. We annotated LF3 and found 4 novel spliced transcripts (RPMS2, RPMS3, RPMS4 and RPMS5) that fully overlap the Ori region, and BILT44 and BIRT21 transcripts of which the 5′-UTR regions overlap the replication origin (Fig. 6b).

### Multigenic transcripts

**Fig. 7:**
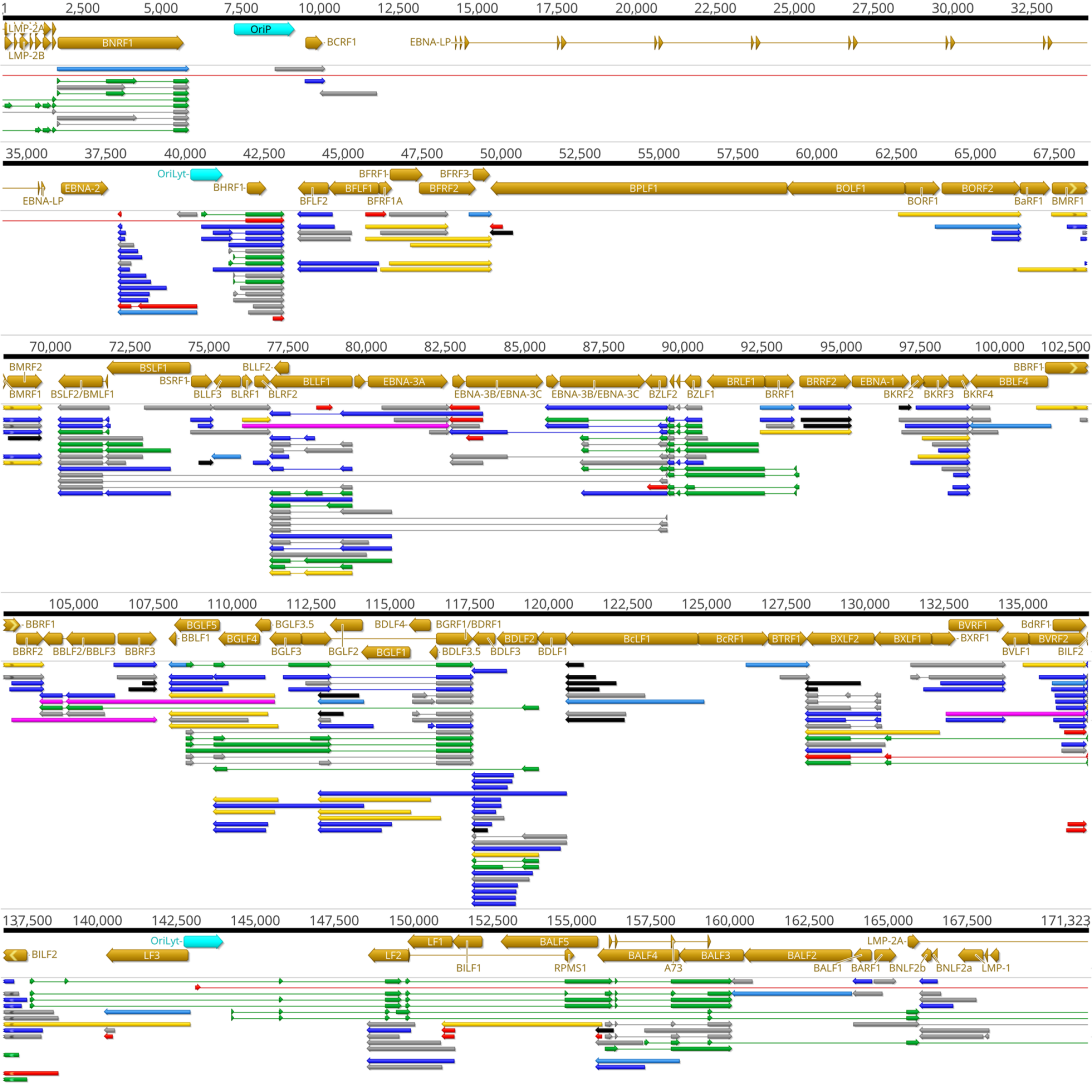
The lytic transcriptome of EBV. Previously detected [26] and novel transcript isoforms are shown on the genome of strain Akata (NCBI accession: KC207813.1). The EBV transcriptome color code: brown arrows: ORFs; aqua rectangles: replication origins; grey: formerly annotated transcripts; light blue: novel monocistronic transcripts; purple: novel polycistronic transcripts; pink: complex transcripts; red: non-coding transcripts; black: 5′-truncated transcripts; dark blue: TSS and TES isoforms. Transcripts generated by the junction region of the circular EBV genome are indicated by asterisk at the genome ends

Multigenic transcripts include bi- and polycistronic mRNA molecules and complex transcripts, the latter contain at least one gene in an opposite orientation relative to the other gene(s). Multigenic transcripts are abundant in every examined large DNA virus, including herpesviruses [35, 36, 42–45]. Multigenic mRNAs have previously been detected in EBV using both SRS [27] and LRS [26] techniques. This study identified forty-seven multigenic transcript of which 27 were novel and demonstrated that basically every lytic gene cluster with the same orientation of ORFs is overlapped by at least one multigenic transcript isoform. Additionally, 4 novel complex transcripts out of altogether 6 complex transcripts (BLRT8, BBRT18, BGLT29 and BVRT9) containing genes with opposite polarity, were also detected. An overview of the transcripts discovered in this study is illustrated in Fig. 7.

### Transcriptional overlaps

We detected all of the 3 forms of transcriptional overlaps including divergent (head-to-head), convergent (tail-to-tail) and tandem (parallel, tail-to-head) overlaps between EBV RNAs. These can be formed between transcripts of adjacent genes, like BDRF1 and BILF2 or long multigenic and monocistronic transcripts, for example BBRT18, a bicistronic transcript overlapping the isoforms of BBTR16 and the isoforms of BBRT14, both in the same orientation and BBRT18, and the isoforms of BGLT29 in the opposite orientation. Several long spliced transcripts also overlap multiple genes. BDLT30 for example initiates upstream of BDLF2 and overlaps the transcript of 12 genes in the same orientation as BDLF2 and the transcript of 3 genes in reverse orientation. Although transcriptional overlaps represent a common phenomenon in EBV, the intergenic regions between the convergent BHRF1 and BHLF1 showed very low level of overlapping transcripts. The intergenic region of BMRF2 and BSLF2/BMLF1 was found to be devoid of transcriptional activity. However, a higher overall transcript coverage may detect a low-level activity at this region.

### Relative transcript abundance

Among the identified transcripts, 198 are relative low abundant, whereas 117 transcripts are moderately and 36 are highly abundant (Additional files 4 and 9). Eleven of the highly abundant transcripts have previously been annotated, while twelve of the new transcripts are short 5′-UTR isoforms, 5 are monocistronic RNAs, 5 are polycistronic transcripts, and 3 are long 5′-UTR isoforms. Fifty-one of moderately expressed transcripts have been previously annotated, while 9 of the new ones are long 5′-UTR isoforms, 27 are short 5′-UTR isoforms, 9 are monocistronic, 9 are polycistronic, 2 are non-coding, and a single one is alternatively terminated, 2 are non-spliced and 6 are putative protein coding transcripts. Forty-eight of low-abundance transcripts have previously been reported, while 18 of the novel transcripts are short 5′-UTR variants, 35 of them are long 5′-UTR isoforms, 1 monocistronic RNAs 13 are polycistronic transcripts, 18 are ncRNAs, 1 is antisense RNA, 4 are complex, 3 are 3′-UTR isoforms, 41 are splice isoforms, 3 are non-spliced isoforms, and 13 are putative protein coding RNAs.

## Discussion

In this study, we report the profiling of the Epstein–Barr virus lytic transcriptome by ONT long-read sequencing platform using amplified and non-amplified cDNA libraries. For the transcript annotation, we used our own dataset as well as SRS and LRS data published previously by other [24–28, 47–49].

These earlier studies have annotated an incomplete lytic transcriptome of EBV. Our multiplatform, integrative approach allowed to obtain a more complete picture on the transcriptomic architecture of this important human pathogen. We identified novel transcripts and RNA isoforms and validated putative transcripts of earlier reports. A total of 241 novel lytic EBV transcripts were detected and 110 previously detected transcripts were confirmed.

A recent study [67] on human adenovirus type 5, a linear dsDNA virus with medium genome size, disclosed a huge plasticity in intron and TES usage. The authors speculate that this flexibility in viral RNA synthesis can lead to selection advantages and thus fuel viral evolution. Previous works on herpesviruses [35, 36, 45, 68, 69] including the EBV [26, 27] uncovered a great variety of transcript length isoforms, the function of which is still mostly unclear. TSS isoforms through uORFs, uATGs and other cis-acting elements of the 5′-UTRs are suggested to play an essential role in translational regulation [70]. Additionally, transcripts with alternative termination may have different turnover times [71, 72], localization [73] and altered translation [74].

In this work, we report the detection of novel length variants and splice isoforms that may alter the coding potential of several viral genes. Further proteomic studies are needed to conclude the potential significance of these transcripts.

We detected a relative large number of short transcript which are embedded into a larger host gene, and contain truncated in-frame ORFs. These transcript type has been described in other viruses, but it turned out that they are more prevalent than it was earlier believed [75].

A number of TSS isoforms was also discovered in this study. The longer transcript variants often contain uORF sequences, which may play a role in the control of translation. We hypothesize that BGLF4 was able to evade translational interruption expected by Watanabe et al. [56] through both its shorter isoforms, or through a complex interplay of uORFs present in the 5′-UTR of its longer isoform.

Here we report the identification of a large number of multigenic transcripts. While polycistronism is a basic property in prokaryotic organisms, it is rare in eukaryotes. The reason for this is that, in prokaryotes, the Shine–Dalgarno sequences allow the translation of each gene in the polycistronic RNA molecules, while in eukaryotic organisms, due to the cap dependent translation initiation, only the most upstream gene of a multigenic transcript is translated. Despite the fact that the viruses of eukaryotes use the same or similar mechanism as their host organisms, they produce a large variety multigenic transcripts, the function of which has not yet been described [76].

A genome-wide antisense expression of the EBV genome has already been described using an SRS approach [24] In this work, we applied an LRS approach that is able to map the transcript ends. According to our results, the majority of antisense transcripts are the results of transcriptional readthroughs between convergent genes or the head-to-head overlap of transcripts encoded by divergently-oriented gene pairs. The question as to whether these transcriptional overlaps are functional, or if so, what their significance is, remains unknown. We have suggested the Transcriptional Interference Network hypothesis [77], which claims that one of their functions is to provide genome-wide gene regulatory mechanism. However, we cannot exclude that the at least a part of these transcriptional overlap represent transcriptional noise without any function. However, the parallel (co-oriented) transcriptional overlaps are not only common, but can be considered as a prototypic design of viral genomes, therefore, we think that other types of overlaps are also functional.

Our analysis also revealed novel Ori-overlapping transcripts. Rennekamp and Lieberman showed in their study [15] that the BHLF1 transcript (overlapping the left Ori-lyt) stably binds to its DNA template, and either BHLF1 or the divergent BHRF1 transcript is necessary for the initiation of lytic replication from this Ori. We detected the TSS and TES of BHLF1 with nucleotide precision, and the existence of a splice isoform of BHLF1 the BHLF2 transcript. We also identified three isoforms of BHRF1, the BHRT15, the BHRT16 and the BHRT17, with a longer than previously detected, Ori-overlapping 5′-UTR. The effect of these novel isoforms on the viral replication is yet to be evaluated. Our research group have identified several Ori-associated transcripts in various viruses [34, 58, 76–78]. We have suggested an interaction between the replication and transcription machineries, which may play a role in the determination of the orientation of the replication fork, and the progression of DNA synthesis [80].

We can raise the question as to whether the low-abundance transcript variants and multigenic RNA molecules are functional, or they represent mere transcriptional noise without contributing to viral proteome or to any function. Further investigations are needed for answering of this question.

Together, we can conclude that multiplatform approaches are important in transcriptomic studies because the different platforms have distinct advantages and limitations, and that they represent independent techniques that are vital for the validation of the results obtained by a particular method.

## Conclusions

This study applies an integrative multi-technique sequencing approach for providing a more complete picture on the transcriptomic architecture of EBV, an important human pathogen. We identified a number of novel transcripts and RNA isoforms, including transcript length and splice variants, and also novel genes embedded into longer host genes containing 5′-truncated in-frame open reading frames, which potentially code for N-terminally truncated proteins. A number of novel non-coding RNAs as well as mono- and multigenic transcripts are also detected.

## Methods

### Cells and viruses

Close to saturation, Akata cells were diluted one-to-one with RPMI-1640 medium supplemented with 10% FCS and pens/strep 24 h before induction. Cells were washed and resuspended to 106 cell/ml in RPMI solution supplemented with Goat anti-human IgG (Jackson, 109-001-003, 17 µg/ml final concentration), or in normal RPMI serving as controls in 7–7 T25 cell culture flasks [24]. 100 µl cell suspensions were aspirated at time points of 10 min, 90 min, 4, 12, 24, 48 and 72 h after resuspension for RNA isolation to verify the success of induction of Epstein–Barr Virus transcription. Applying real-time PCR the activity of the BZLF and GP350 genes were monitored normalized to reference genes. The remaining cells were pelleted and stored at − 70 °C.

### Assessment of the lytic induction

Total RNA was isolated from 100 µl cell suspensions with Direct-zol method (Zymo) as recommended by the manufacturer. DNase I treatment (Thermo, EN0525) was performed subsequently according to the manufacturer. RNA samples were amplified in a one-step reaction using oligonucleotides specific for BZLF, GP350 transcripts and ACTB and GADPH as reference genes (Table 2) with the SYBR-Green based approach (Bioline, SensiFAST™ SYBR® No-ROX One-Step Kit, BIO-72001) as recommended in the manual as follows: reverse transcription: 50 °C, 10 min; Initial denaturation: 95 °C 2 min, 45 cycles of 95 °C, 10 s and 60 °C, 30 s. Amplification specificity was checked with melting curve analysis.

**Table 2:** Primers used for the assessment of lytic induction

Primer name	Sequence
BZLF1 F	CCCAAACTCGACTTCTGAAGATGTA
BZLF1 R	TGATAGACTCTGGTAGCTTGGTCAA
gp350 F	AGAATCTGGGCTGGGACGTT
gp350 R	ACATGGAGCCCGGACAAGT
ACTB fw	GGCGGCACCACCATGTACCCT
ACTB rv	AGGGGCCGGACTCGTCATACT
GAPDH fw	GGAAGGTGAAGGTCGGAGTCA
GAPDH rv	ATGGGTGGAATCATATTGGAACA

### RNA isolation for sequencing

Total RNA was purified from the cells using the NucleoSpin RNA Kit (Macherey–Nagel). Total RNA samples were split in two. Polyadenylated RNAs were isolated from half of the total RNA samples using the Oligotex mRNA Mini Kit (Qiagen). Ribodepletion, was carried out to remove ribosomal RNA from the other half of total RNAs using Epicentre Ribo-Zero Magnetic Kit. The concentrations of RNA samples were determined using Qubit 4 (Thermo Fisher Scientific). The RNA BR Assay Kit (Thermo Fisher Scientific) was used for the quantification of total RNAs while the Qubit RNA HS Assay (Thermo Fisher Scientific) Kit was applied for the measurement of polyadenylated and ribodepleted samples. The RNA quality was measured with a TapeStation 4150 (Agilent).

### Amplified cDNA library preparation

Amplified cDNA libraries were prepared from the purified polyA(+) RNAs using ONT Ligation Kit 1D (SQK-LSK109). Briefly, 50 ng of polyA selected RNA was reverse transcribed using SuperScript IV Reverse Transcriptase (Thermo Fisher Scientific) and (VN)T20 (oligo dT) primers (from the ONT kit). The cDNA samples were amplified using Kapa HiFi PCR Kit (Kapa Biosystems), followed by end repair treatment [NEBNext End repair/dA-tailing Module (New England Biolabs)]. The libraries were barcoded using 1D PCR Barcoding (96) Kit (ONT) following the manufacturer’s instructions. Between each step the samples were purified using Agencourt AMPure XP magnetic beads (Beckman Coulter). The concentration of the libraries was determined by Qubit 4 Fluorometer. Barcoded libraries were pooled in equimolar ratios and 200 fmol of the pooled sample was loaded on a MinION Flow Cell.

Amplified cDNA library was also generated from 50 ng ribodepleted RNA and custom-made random primers. The consecutive steps were the same as described above.

### Non-amplified cDNA library preparation

ONT Direct cDNA Sequencing Kit (SQK-DCS109) was used for the generation of amplification-free libraries. In short, 100 ng polyA-selected RNA sample was used for the synthesis of the first cDNA strand using Maxima H Minus Reverse Transcriptase (Thermo Fisher Scientific) RNase Cocktail Enzyme Mix (Thermo Fisher Scientific) was used for the removal of RNAs from the single stranded cDNA molecules. The synthesis of the second cDNA strand was performed using LongAmp Taq Master Mix (New England Biolabs). cDNA ends were repaired using NEBNext Ultra II End Repair/dA-Tailing Module. The cDNA ends were repaired using NEBNext Ultra II End Repair/dA-Tailing Module (New England Biolabs). Libraries were barcoded using ONT Native Barcoding Expansion Kit (EXP-NBD104), then the ligation of the sequencing adapter was carried out using NEB Quick T4 DNA Ligase. All conditions were set according to the SQK-DCS109 manufacturer’s protocol.

### Pre-processing and data analysis

MinION data were base-called and demultiplexed using Guppy base caller v. 3.3.3. with –qscore_filtering turned on. Reads with a Q-score larger than 7 were mapped to the circularized viral genome (NCBI nucleotide accession: KC207813.1) using the Minimap2 software [81]. Adapter sequences and poly(A) tails were preserved on reads to determine 5′ and 3′ ends and the orientation of the transcripts. Previously published [24–28, 47–49] CAGE-Seq, PA-seq, Illumina and PacBio RSII data were retrieved for TSS, TES, intron and transcript validation.

### Isoform annotation

#### Analysis of transcript features obtained from long-read sequencing

Transcript isoform detection and annotation was carried out using the LoRTIA software suite v.0.9.9 [https://github.com/zsolt-balazs/LoRTIA] as follows: (1) artefacts resulting from false priming or partial RT or PCR were removed by searching non-trimmed read ends for the sequencing adapters for the TSS or a homopolymer A sequence for the TES. The first nucleotide not aligning with the adapter or the homopolymer A was denoted as possible TSS or TES. Any other read ends were excluded from further analysis; (2) Random start and end positions caused by RNA degradation were further filtered by testing the putative TSSs and TESs against the Poison distribution, with the significance corrected by the Bonferroni method [82]. Features failing to qualify as local maxima, or being present in less than 1‰ of the coverage were eliminated from the analysis; (3) Gaps were denoted as putative introns, if they have one of the three most frequent consensus sequence (GT/AG, GC/AG, AT/AC) and if they are more abundant than 1‰ compared to the local coverage. Putative introns flanked by tandem repeat regions were removed from the analysis as possible template-switching artefacts.

The LoRTIA suit was set as follows: (1) for oligo(dT)-primed cDNA reads: − 5 TGCCATTAGGCCGGG –five score 16 – check_in_soft 15 3 AAAAAAAAAAAAAAA –three score 16-spoisson–f True; (2) for dcDNA-seq reads: − 5 GCTGATATTGCTGGG—five score 16—check_in_soft 15—3 AAAAAAAAAAAAAAA—three_score 16 -spoisson -f True.

#### Analysis of transcript features obtained from Illumina sequencing

The raw Illumina reads were trimmed with the Trimgalore. (https://www.bioinformatics.babraham.ac.uk/projects/trim_galore/) The above-mentioned EBV reference genome was indexed using STAR aligner v2.7.3a [83] using the following settings: –genomeSAindexNbases 7, followed by the mapping of the reads with default options. STAR software was also used to detect introns from the SRS samples.

Bam files obtained from CAGE-seq were converted to BigWig format to detect 5′ end coverage. The CAGEfightR (R/Bioconductor) package [84] was used to determine TSS positions. The TSS clusters within a 10 nucleotides window were termed identical. Clusters with a “minimum pooled value” (--pooledcutoff) of 0.1 and below were excluded from the further analysis. Then, the cluster positions with a score of 25 or lower were filtered out.

The same approach was used for the TES identification, with the following exception: “minimum pooled value” was set to 10.

#### Annotation of transcript features obtained from LRS and SRS techniques

Putative TSSs and TESs were accepted as real if presented in either two of our techniques, or for TSSs in one of our techniques and one in either CAGE-Seq or PacBio results, for TESs in one of our techniques and either PA-Seq or the PacBio results. Likewise, putative introns were accepted as real if they were present in two of our techniques, or in one of our techniques and the PacBio results.

#### Annotation of transcripts

Accepted TSSs, TESs and introns were assembled into transcripts using the Transcript_Annotator software of the LoRTIA toolkit. Very long unique or low-abundance reads which could not be detected using LoRTIA were evaluated and annotated manually. These reads were also accepted as putative transcript isoforms if they were longer than any other overlapping RNA molecule.

### Transcript nomenclature

We used the conventional terminology for naming the EBV transcriptome [26]. Novel transcript isoforms were named after the most abundant previously annotated transcript of a gene.

### Coding potential estimation

In order to estimate coding potential of the transcripts with previously undetected ORFs, we extracted the transcript sequences from the reference genome and used the Coding-Potential Assessment Tool (CPAT) [61] with default settings.

## Supplementary Information

**Additional file 1.** Detailed descriptive statistics of the sequencing datasets. (A) Oxford Nanopore; B (Illumina).

**Additional file 2.** TSSs of EBV. TSSs were detected using the LoRTIA software. The -150–0 nt region upstream from the TSS was analyzed for GC, CAAT and TATA boxes. Distances from the TSS are given in nucleotides. The ±10 nts region of the TSS was also retrieved for sequence analysis. The TSS positions from previous CAGE-Seq [26] and PacBio [26] analyses were matched with our results. The sample number in which a given TSS was detected is shown. TSS coordinates are according to the genome of strain Akata (NCBI accession: KC207813.1).

**Additional file 3.** TESs of EBV. TESs were detected using the LoRTIA software. The -50–0 nt region upstream from the TES was analyzed for PASs. Distances from the TES are in nucleotides. The ±50 nts region of the TES was also retrieved for sequence analysis. The TES positions from previous PE-seq experiment was matched with our results. The sample number in which a given TES was detected is shown. TES coordinates are according to the genome of strain Akata (NCBI accession: KC207813.1).

**Additional file 4.** Transcripts of EBV. The transcripts were annotated using the LoRTIA software. The transcript coordinates are according to the genome of strain Akata (NCBI accession: KC207813.1). Transcript abundance in each sample is also shown.

**Additional file 5.** The list of the transcripts determined in our study and the comparison of our data with those of published by O’Grady and colleagues.

**Additional file 6.** Putative transcripts of EBV. Unique very long reads were named putative transcripts, because of their uncertain TSS position. Transcript coordinates are according to the genome of strain Akata (NCBI accession: KC207813.1).

**Additional file 7.** The introns of EBV. The introns were detected using the LoRTIA software. Read counts per sample are shown. Splice junction coordinates are according to the genome of strain Akata (NCBI accession: KC207813.1).

**Additional file 8.** Coding potential and BLAST analysis of alternatively spliced transcripts. For the analysis of the coding potential we used the CPAT tool.

**Additional file 9.** Abundance of EBV transcripts. Color code. Light grey: low abundance: 1–10 reads, dark gray: medium abundance: 11–100 reads, black—high abundance: 101–3000 reads. See Additional file 4 for more details.

## Data Availability

The LoRTIA software suite is available on GitHub: https://github.com/zsolt-balazs/LoRTIA. Our in-house scripts used to generate the descriptive statistics of reads and transcripts, to analyze promoters and to detect transcript isoforms are also available on GitHub: https://github.com/moldovannorbert/seqtools. The sequencing datasets generated during this study are available at the European Nucleotide Archive’s SRA database under the accession PRJEB38992: (https://www.ebi.ac.uk/ena/browser/view/PRJEB38992).

## Abbreviations

EBV: Epstein–Barr virus
CPAT: Coding-potential assessment tool
duORFs: Disruption of upstream open reading frame
IM: Infectious mononucleosis
LCLs: Lymphoblastoid cell lines
lncRNAs: Long noncoding RNAs
LRS: Long-read sequencing
ncRNAs: Non-coding RNAs
ONT: Oxford nanopore technologies
PAS: Polyadenylation signals
SRS: Short-read sequencing
TES: Transcriptional end sites
TS: Template-switching
TSS: Transcriptional start sites
uORFs: Upstream ORFs
uAUGs: Upstream AUGs
